# ICTV Virus Taxonomy Profile: Lispiviridae 2023

**DOI:** 10.1099/jgv.0.001869

**Published:** 2023-07-11

**Authors:** Jun-Min Li, Fei Wang, Gongyin Ye, Sofia Paraskevopoulou

**Affiliations:** 1 Institute of Plant Virology, Ningbo University, Ningbo, PR China; 2 Wuhan Institute of Virology, Chinese Academy of Sciences, Wuhan, PR China; 3 Institute of Insect Sciences, Zhejiang University, Hangzhou, PR China; 4 Genome Competence Center (MF1), Robert Koch Institute, Berlin, Germany

**Keywords:** ICTV report, *Lispiviridae*, taxonomy

## Abstract

Members of the family *Lispiviridae* are viruses with negative-sense RNA genomes of 6.5–15.5 kb that have mainly been found in arthropods and nematodes. The genomes of lispivirids contain several open reading frames, typically encoding a nucleoprotein (N), a glycoprotein (G), and a large protein (L) including an RNA-directed RNA polymerase (RdRP) domain. This is a summary of the International Committee on Taxonomy of Viruses (ICTV) Report on the family *Lispiviridae*, which is available at ictv.global/report/lispiviridae.

## Virion

Unknown.

## Genome

**Fig. 1.**
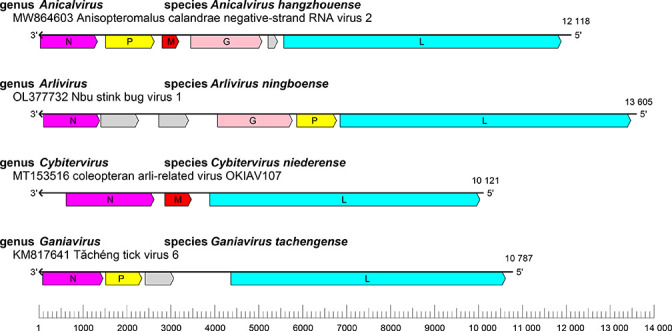
Genome organization of representative viruses of the family *Lispiviridae*. The diagram shows the genome length, position of open reading frames and putative protein products: N, nucleoprotein (magenta); P, predicted phosphoprotein (yellow); M, predicted matrix protein (red); G, glycoprotein (salmon); L, large protein (cyan) including an RNA-directed RNA polymerase domain; predicted protein of unknown function (grey).

The genomes of lispivirids are single molecules of linear, negative-sense RNA of 6.5–15.5 kb (Table 1). Lispivirid genomes typically contain five to six open reading frames (ORFs). For example, the genome of Anisopteromalus calandrae negative-strand RNA virus 2 (species *Anicalvirus hangzhouense*, genus *Anicalvirus*) has six ORFs encoding a putative nucleoprotein (N), predicted phosphoprotein (P), predicted matrix protein (M), glycoprotein (G), unknown protein (X) and L protein (L) including an RNA-directed RNA polymerase (RdRP) domain; these ORFs are organized in the order 3′-N-P-M-G-X-L-5′ (Fig. 1) [1]. It should be noted that the genomes of some lispivirids lack one or more of these genes, which might be due to coding incompleteness.

**Table 1.** Characteristics of members of the family *Lispiviridae*

Example	Anisopteromalus calandrae negative-strand RNA virus 2 (MW864603), species *Anicalvirus hangzhouense*, genus *Anicalvirus*
Virion	Unknown
Genome	6.5–15.5 kb of negative-sense RNA
Replication	Unknown
Translation	Unknown
Host range	Arthropods and nematodes of the superphylum Ecdysozoa
Taxonomy	Realm *Riboviria*, kingdom *Orthornavirae*, phylum *Negarnaviricota*, class *Monjiviricetes*, order *Mononegavirales*: >20 genera and >25 species

## Replication

Unknown.

## Taxonomy

**Fig. 2.**
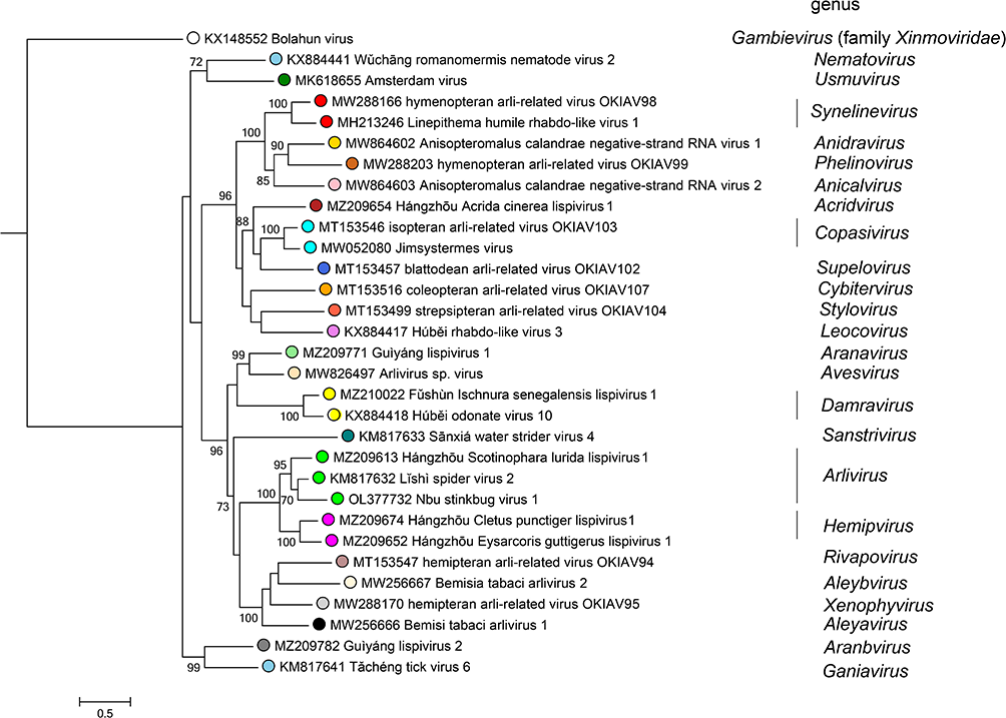
Phylogenetic relationships of lispivirids. The phylogenetic tree is based on a mafft-alignment of the RdRP amino acid sequences using the l-INS-i algorithm and was inferred using ModelTest-NG and the LG substitution model. Numbers on branch nodes represent transfer bootstrap expectation values (1000 replicates). The tree is rooted to Bolahun virus of the *Gambievirus* genus of the *Xinmoviridae* family.

Current taxonomy: ictv.global/taxonomy. The family *Lispiviridae* includes >20 genera and >25 species (Fig. 2) for viruses hosted by arthropods and nematodes of the superphylum *Ecdysozoa* [1,6]. Lispivirids have been detected in mammals and bird faeces [7,8], but it is still unclear whether they are capable of replicating in these hosts.

## Resources

Full ICTV Report on the family *Lispiviridae*: ictv.global/report/lispiviridae.

## Abbreviations

G: glycoprotein
L: large protein
M: matrix protein
N: nucleoprotein
P: phosphoprotein
RdRP: RNA-directed RNA polymerase
X: protein of unknown function

## References

[1] Wang F, Yuan B, Xiao S, Zhang J, Jia W (2021). Diverse RNA viruses discovered in three parasitoid wasps of the rice weevil *Sitophilus oryzae*. mSphere.

[2] Huang H-J, Ye Z-X, Wang X, Yan X-T, Zhang Y (2021). Diversity and infectivity of the RNA virome among different cryptic species of an agriculturally important insect vector: whitefly *Bemisia tabaci*. NPJ Biofilms Microbiomes.

[3] Käfer S, Paraskevopoulou S, Zirkel F, Wieseke N, Donath A (2019). Re-assessing the diversity of negative strand RNA viruses in insects. PLoS Pathog.

[4] Lay CL, Shi M, Buček A, Bourguignon T, Lo N (2020). Unmapped RNA virus diversity in termites and their symbionts. Viruses.

[5] Viljakainen L, Holmberg I, Abril S, Jurvansuu J (2018). Viruses of invasive Argentine ants from the European Main supercolony: characterization, interactions and evolution. J Gen Virol.

[6] Ye Z-X, Wang S-M, Lu G, Chen J-P, Zhang C-X (2022). Complete genome sequence of a novel arlivirus from a yellow spotted stink bug (*Erthesina fullo* (Thunberg, 1783)). Arch Virol.

[7] Williams SH, Che X, Oleynik A, Garcia JA, Muller D (2019). Discovery of two highly divergent negative-sense RNA viruses associated with the parasitic nematode, *Capillaria hepatica*, in wild *Mus musculus* from New York City. J Gen Virol.

[8] Zhu W, Yang J, Lu S, Jin D, Pu J (2022). RNA virus diversity in birds and small mammals from Qinghai–Tibet Plateau of China. Front Microbiol.

